# Cucurbitacin E reduces obesity and related metabolic dysfunction in mice by targeting JAK-STAT5 signaling pathway

**DOI:** 10.1371/journal.pone.0178910

**Published:** 2017-06-09

**Authors:** Munazza Murtaza, Gulnaz Khan, Meha Fatima Aftab, Shabbir Khan Afridi, Safina Ghaffar, Ayaz Ahmed, Rahman M. Hafizur, Rizwana Sanaullah Waraich

**Affiliations:** Dr. Panjwani Center for Molecular Medicine and Drug Research, International Center for Chemical and Biological Sciences, University of Karachi, Karachi, Pakistan; Universidad Pablo de Olavide, SPAIN

## Abstract

Several members of cucurbitaceae family have been reported to regulate growth of cancer by interfering with STAT3 signaling. In the present study, we investigated the unique role and molecular mechanism of cucurbitacins (Cucs) in reducing symptoms of metabolic syndrome in mice. Cucurbitacin E (CuE) was found to reduce adipogenesis in murine adipocytes. CuE treatment diminished hypertrophy of adipocytes, visceral obesity and lipogenesis gene expression in diet induced mice model of metabolic syndrome (MetS). CuE also ameliorated adipose tissue dysfunction by reducing hyperleptinemia and TNF-alpha levels and enhancing hypoadiponectinemia. Results show that CuE mediated these effects by attenuating Jenus kinase- Signal transducer and activator of transcription 5 (JAK- STAT5) signaling in visceral fat tissue. As a result, CuE treatment also reduced PPAR gamma expression. Glucose uptake enhanced in adipocytes after stimulation with CuE and insulin resistance diminished in mice treated with CuE, as reflected by reduced glucose intolerance and glucose stimulated insulin secretion. CuE restored insulin sensitivity indirectly by inhibiting JAK phosphorylation and improving AMPK activity. Consequently, insulin signaling was up-regulated in mice muscle. As CuE positively regulated adipose tissue function and suppressed visceral obesity, dyslipedemia, hyperglycemia and insulin resistance in mice model of MetS, we suggest that CuE can be used as novel approach to treat metabolic diseases.

## Introduction

According to an estimate a quarter of the world’s population is suffering from metabolic syndrome (MetS) [1]. Central obesity is associated with resistance to effects of insulin in the periphery such as utilization of glucose and fatty acid [2]. Additionally associated factors of insulin resistance such as hyperinsulinmia, hyperglycemia, and cytokine/adipokine production can lead to abnormal lipid profile, endothelial dysfunction, vascular inflammation and hypertension [3, 4]. Interestingly, individuals with abdominal obesity but normal weight also exhibit a similar profile [5]. Therefore, there is a need to address metabolic risk factors in order to reduce morbidity and mortality associated with cardiovascular diseases and diabetes. Currently, inhibitors of pancreatic lipases are the only drugs for long term treatment of obesity [6]. Also, current obesity drugs exhibit hazardous side effects [7]. Thus, there is a needfor development of new drug targets. The JAK-STAT signaling pathway occurs in all cells; however, this pathway can mediate cell specific responses. Current evidence supports the role of JAK-STAT signaling in adipose tissue function [8, 9] such as adipose tissue development and physiology. STAT target genes in adipocytes reveal how these transcription factors impact various areas of adipocyte metabolism including insulin action, modulation of lipid stores, and glucose homeostasis [10].

**Fig 1:**
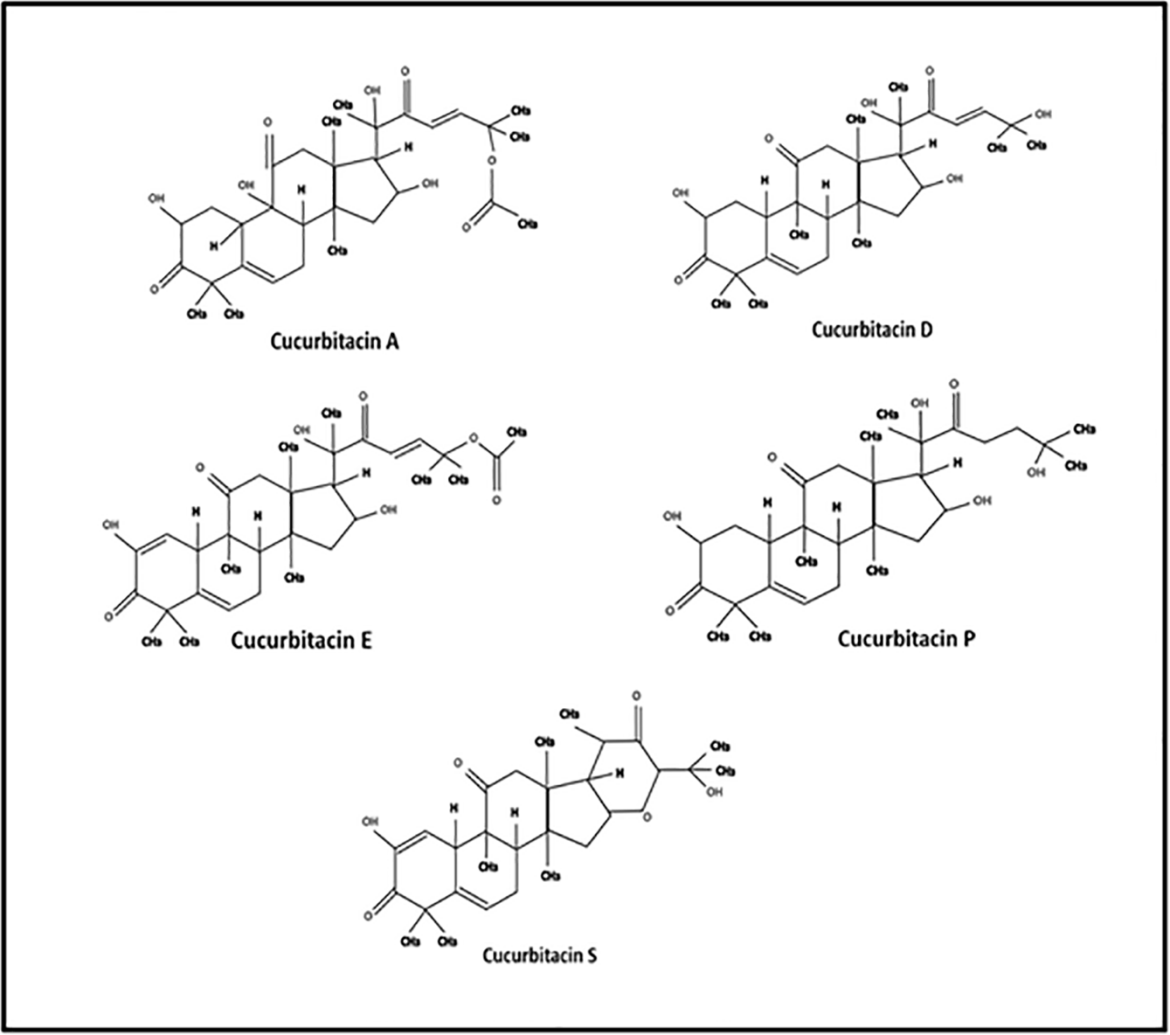
Structure of cucurbitacins.

Cucurbitacin family members found in cucumber, melon, watermelon, squash, and pumpkin have a range of biological and pharmacological activities [11]. Current evidence indicates that Cucs has growth inhibitory effects on several cancer cells such as bladder cancer, pancreatic cancer, hepatocarcinoma, breast cancer and leukemia [12, 13]. Reports also indicate that cucurbitacins inhibit JAK-STAT signaling in several cancer cell lines [14, 15]. A recent study showed inhibitory role of cucurbitacin I and B on adipocytes *in vitro* [16]. However, the role of cucurbitacins in reducing obesity and related metabolic complications in mice has not been investigated yet. Therefore, we hypothesized that cucrbitacins may reduce obesity related metabolic complications by targeting JAK-STAT signaling pathway. The name and structures of the cucurbitacins selected for the present study are shown in, Fig 1A.

## Materials and methods

### Cell lines, reagents, and antibodies

3T3-L1 pre-adipocytes were purchased from ATCC (Virginia, USA). Fetal calf serum (South American origin) was sourced from Biowest. The cell culture media and supplements were acquired from Gibco (Sigma, St.Louis, USA). Antibodies against phospho-Jak2 (Tyr1007/1008), phospho-Stat5a (Tyr694), phospho-AMPKα (Thr172), phospho serine 473 of Akt, the total Akt protein were obtained from Cell Signaling Technology (Beverly, MA, USA). Phospho serine 307 of IRS-1 and the total IRS-1 protein antibody were procured from Millipore, cucurbitacins from Sigma, St.Louis, USA, and Glucose-6-phosphate dehydrogenase (G6PDH) from MP Biomedicals, France.

### Animals

C57BL/6 male mice were purchased from Jackson lab (Maine, USA). Mice were housed in standard environment (22+ 2°C, 40–70% humidity and 12 hr light and dark cycle). All animal experiments were approved by the Animal Care and Use Committee of the International Centre for Chemical and Biological Sciences and were conducted in accordance with the Guide for the Care and Use of Animals published by the National Institutes of Health. The metabolic syndrome mice model was developed by feeding mice a high fat diet as described previously [17]. The mice were designated as metabolic syndrome mice (HFD-MetS-mice). Briefly, the mice were randomly assigned into two groups according to their diet for 8 weeks (n = 10–12): high fat diet group (HFD) (60% fat, 20% carbohydrate, 20% protein by Mucedola, ETPF1916) or the matched low fat, standard diet group (SD) (10% fat, 70% carbohydrate, 20% protein, by Mucedola, ETPF1920). After eight weeks on high fat diet, the mice with significant obese phenotype and fasting blood glucose levels ≥ 126 mg/dl were considered MetS mice. The MetS mice were continued on the HFD throughout the study. The MetS mice were then randomly divided into three additional groups, according to the treatment administered by oral gavage for 10 weeks (n = 10–12): a low dose 0.25mg/kg/day of cucurbitacin E designated as, HFD+ CuE (L) or high dose 0.5 mg/kg/day of cucurbitacin E, designated as HFD+CuE (H) or 50 mg/kg/day Orlistat (HFD+Orlistat). Cucurbitacin and orlistat were dissolved in 0.5% carboxymethylcellulose (CMC). Animals on SD were administered 0.5% CMC by oral gavage.

### Cell culture and cell lysis

3T3-L1 pre-adipocytes were cultured in Dulbecco’s modified Eagle’s medium containing 25 mM glucose, 10% fetal calf serum, 2 mM glutamine. Cells were stimulated to differentiate with DMEM 3-isobutyl-1-methylxanthine (IBMX, 0.5 mM), dexamethasone (1 μM), and insulin (10 μg/mL) as described previously [18]. For *in vitro* experiments, 3T3-L1 adipocytes were lysed with lysis buffer (50 mM HEPES (pH 7.6), 150 mM NaCl, 1% Triton X-100, protease inhibitors and phosphatase inhibitors). For *in vivo* investigation of insulin signaling in muscle tissue, mice were injected with 5 IU insulin into the inferior vena cava. Fat and skeletal muscle tissues were removed and homogenized at 4°C (Stuart Homogeniser, UK) in lysis buffer. The tissues were then processed as described previously [19]. Briefly, homogenates were solubilized on ice for 30 min followed by centrifugation at 12,000 × g for 15 min. The supernatant was separated and protein content was determined by the Bradford method. Fifty micrograms of the total protein was separated by SDS-PAGE (7.5%) and western blot analysis was performed as previously described [20].

### Oil red O staining

Fully differentiated, 3T3-L1 adipocytes were washed twice with PBS and were fixed in 10% formalin for 1 hour at room temperature. After fixation, formalin was removed and cells were washed with sterile water. Cells were then stained in 0.5% oil red O solution and incubated at 37°C for 30 minutes followed by 4–5 times washing with sterile water. Images were taken under a microscope.

### Biochemical analysis

Serum, LDL and cholesterol were determined by HDL/LDL cholesterol assay kit from abcam (MA, USA). Free fatty acid was measured in serum using FFA assay kit from Cell Biolabs Inc. (San Diego, CA, USA). Serum and cellular TG contents were determined by triglyceride colorimetric kit (Cayman chemicals, MI, USA) as directed by the manufacturers.

### Enzyme-linked immunosorbent assay (ELISA)

Blood adiponectin, leptin (Crystal Chem, IL, USA) and TNF-alpha (Millipore, MA, USA) levels were measured by their respective mouse ELISA based kits according to the manufacturer’s instructions.

### RT-PCR

Using Quantitative PCR (Startagene MX 3000P, Agilent technologies, Germany) the mRNA expression levels were determined. mRNA expression levels in different tissues were normalized to beta-actin and quantified as described [21]. Briefly, RNA was extracted from the tissues using Trizol Reagent (Invitrogen, CA, USA), 1 microgram RNA was used for cDNA synthesis using revertAid first strand cDNA synthesis kit (Thermoscientific, Louisiana, EU). Primer sequences are available upon request.

### Glucose uptake assay

Following differentiation, adipocytes were cultured in Dulbecco’s modified eagle’s medium with 10% fetal calf serum, 100 units/ml penicillin, 100 μg/ml streptomycin, 25 mM glucose and 2 mM glutamine. Adipocytes were stimulated with TNF-alpha and cucurbitacins, for 24 hours. After stimulation with insulin, fat cells were incubated with 1 mM 2DG. 2DG6P uptake by adipocytes was measured by an enzymatic fluorescence assay as previously described [22].

### Histology of visceral white adipose tissues

Perigonadal fat tissue was collected from all groups of mice followed by fixation in 10% paraformaldehyde. The tissues were embedded in paraffin and were further processed for the hematoxilin and eosin staining as described previously [23].

### Glucose tolerance test (GTT)

The mice were fasted for 14–16 hours before GTT. Their blood glucose levels were measured at 0, 15, 30, 90 and 120 min after glucose injection (2 g/kg body weight), as previously described. [24]. The plasma glucose levels were measured with an Onetouch Ultra glucometer (LifeScan Inc., USA). The results were expressed as area under the curve (AUC 0–120 min).

### Glucose-stimulated insulin secretion (GSIS)

After an overnight fast, the mice were injected with 2 g/kg body weight of glucose [25]. To determine the plasma insulin levels in response to glucose, blood samples were collected from the tail vein at 0, 30 and 60 min after glucose injection. The collected samples were separated by centrifugation at 4°C and stored at -20°C until assay. The plasma insulin levels were measured using an ELISA kit (Crystal Chem, IL, USA).

### Statistical analysis

Results were derived from at least five independent experiments. After calculating mean ± SEMs, groups of data were compared either using one-way ANOVA, followed by post hoc analysis (using Dunnett’s multiple comparison tests), or two way ANOVA followed by post hoc analysis (using Bonferroni test). A p-value ≤ 0.05 was considered to be statistically significant.

## Results

### Effect of cucrbitacins on adipgenesis and insulin resistance *in vitro*

**Fig 2:**
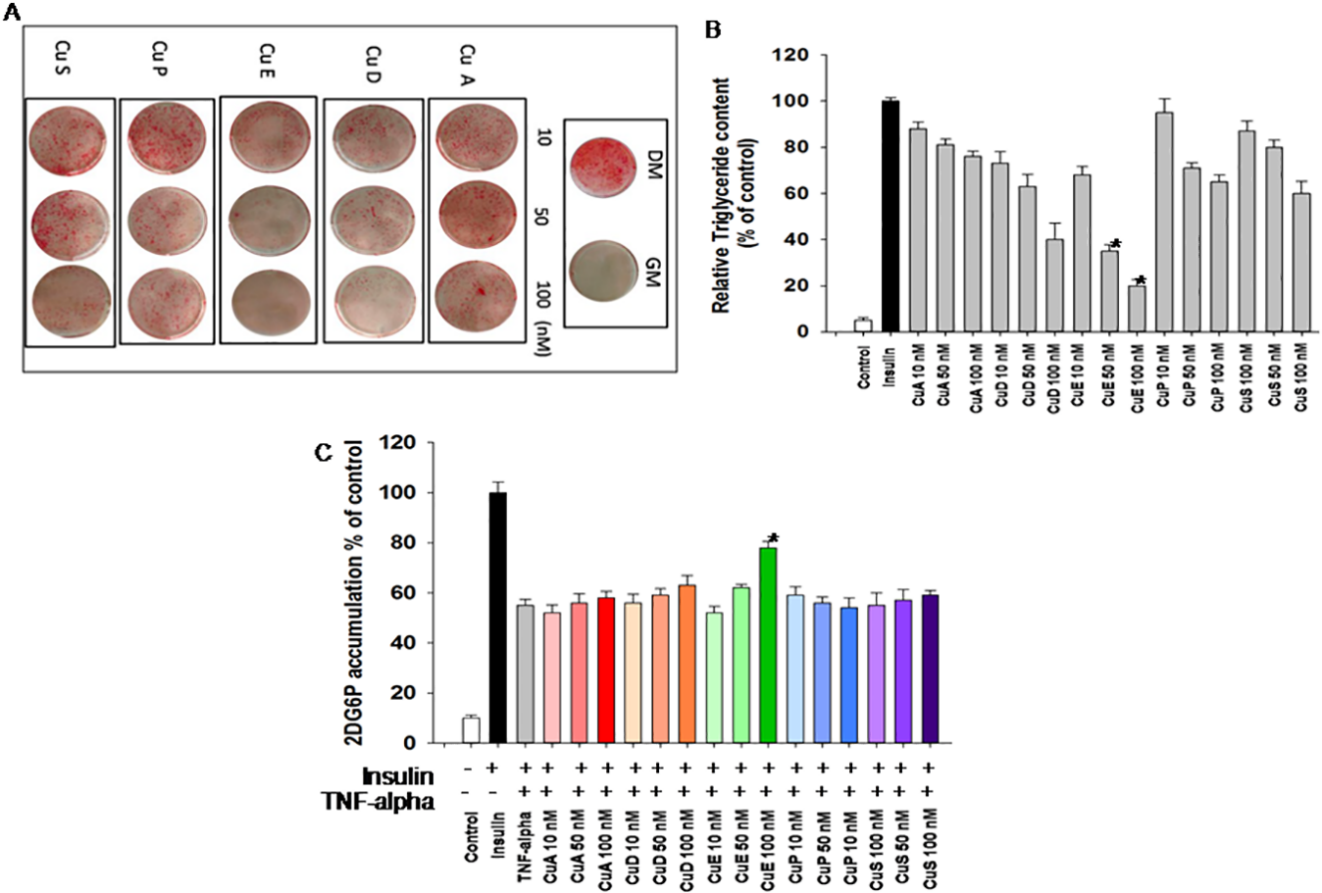
Effect of cucurbitacins on adipogenesis. (A) Oil red O staining in 3T3-L1 preadipocytes differentiated into adipocytes and treated with different concentrations of cucurbitacins. GM, growth media, DM, differentiation media. (B) Quantification of cellular TG content. Cellular triglyceride content is relative to cells treated with DM alone (deemed 100%). n = 4–5 independent experiments, results represent mean ± SEMs. *P < 0.05, cucurbitacins vs. cells treated with DM alone. (C) Glucose uptake in adipocytes. Differentiated 3T3-L1 adipocytes were incubated with 20ng/ml TNF-alpha and CuE for 24 hours followed by stimulation with 10nM insulin for 1 hour. Results are mean ± SEMs of five experiments, *P < 0.05. cucurbitacins vs. cells treated with TNF-alpha alone.

Cucurbitacins (CuA, CuD, CuE, CuP, CuS) were first evaluated for their effect on differentiation of 3T3-L1 mouse embryo fibroblasts into mature adipocytes, in dose dependent manner. We observed that cucurbitacin E (CuE) significantly reduced adipocyte formation as compared to cells treated with differentiation medium alone (Fig 2A). We also observed that these results were reflected in intracellular triglyceride content, in dose dependent manner (Fig 2B). Furthermore, potential effect of cucurbitacins (Cucs) on insulin action in fat cells was determined by measuring glucose uptake in TNF-alpha mediated insulin resistant adipocytes (Fig 2C). Cells treated with Cu E indicated enhanced glucose uptake in mature adipocytes. Earlier, we selected the dose of Cucs that did not affect cell viability (supporting information, S1 Fig).

### Effect of cucurbitacin E on body weight and visceral obesity in diet induce mice model of metabolic syndrome

**Fig 3:**
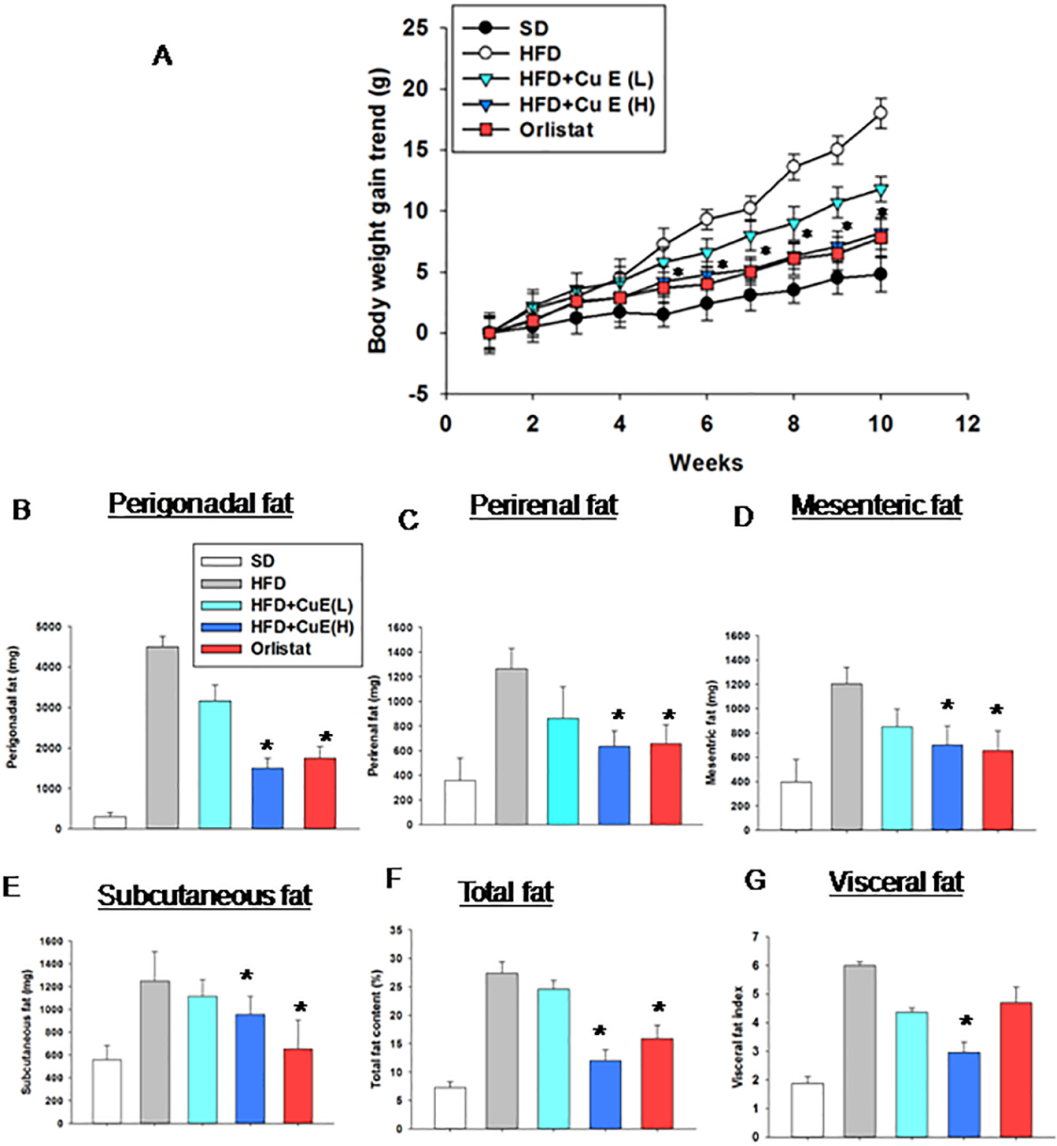
Effect of cucurbitacin E on body fat content (A) Comparative weekly body weight gain trends in SD, HFD, HFD and CuE (L) 0.25mg/kg, HFD and CuE (H) 0.5mg/kg and HFD+Orlistat mice for 10 weeks (n = 12–15). (B) Proportion of perigonadal, (C) perirenal (D), mesenteric (E) and subcutaneous fat pads weights. (F) Proportion of total body fat content.(G) Visceral fat index. n = 10–12 in each group, results represent means ± SEMs. *P < 0.05, mice treated with CuE or Orlistat vs HFD-MetS mice.

We developed a high fat diet mice model of metabolic syndrome (HFD-MetS) [17] to assess the role of CuE on body weight and fat tissue biology. We found significant decrease in body weights of HFD-MetS mice treated with CuE (0.5mg/kg) as compared to HFD-MetS mice treated with vehicle alone (Fig 3A). CuE treatment reduced all fat pads weights in HFD-MetS mice (Fig 3B, 3C, 3D and 3E). We observed 55% reduction in total fat in mice, after treatment with CuE in comparison to HFD-MetS mice (Fig 3F). Abdominal obesity is strongly associated with metabolic syndrome [26]. Interestingly, central obesity was reduced to 50% after CuE treatment as compared to HFD MetS mice (Fig 3G), elucidating the effectiveness of CuE in targeting MetS.

### Determination of role of CuE on adipose tissue biology

**Fig 4:**
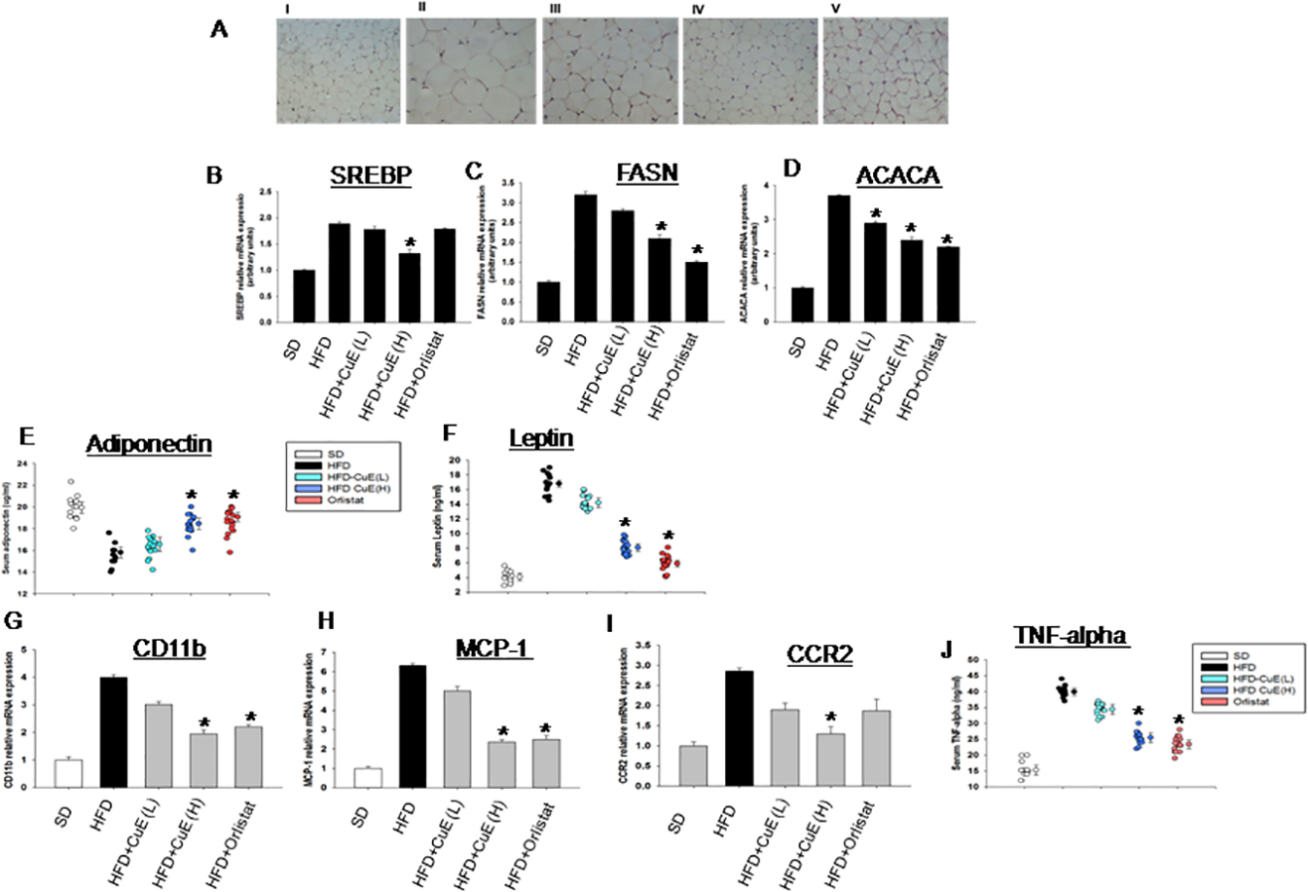
Effect of CuE on adipose tissue morphology and function. (A) H and E stained tissue of perigonadal fat from (I) SD (II) HFD (III) HFD+CuE (L) (IV) HFD+CuE (H); and (V) HFD+Orlistat mice. (B-D) Lipogenic gene expression of SREBP, FASN and ACACA genes was measured in visceral fat tissue, by quantitative PCR. (E) Serum adiponectin concentration. (F) Serum leptin levels were measured by ELISA. (G-I) Macrophage infiltration and recruitment gene expression of CD11b, MCP-1 and CCR2 genes was measured in visceral fat tissue, by quantitative PCR. *P < 0.05 HFD-MetS mice treated with CuE or Orlistat vs HFD mice model of MetS. Results are mean ± S.E. (n = 12–15). (J) Serum TNF-alpha concentration.

In order to evaluate the role of CuE in adipose tissue function, we examined hypertrophy in visceral fat of mice. We found that CuE treated mice showed diminished hypertrophy induced by high fat diet (Fig 4A). CuE also reduced lipogenesis in these mice as reflected by attenuation of mRNA expression of transcriptional factors such as Sterol Regulatory Element-Binding Protein *(SREBP)*, also enzymes: Fatty Acid Synthase *(FASN)* and Acetyl-CoA Carboxylase Alpha *(ACACA)* (Fig 4B, 4C and 4D). Metabolic syndrome results as direct deregulation of adipokines release in the blood [27]. We observed that adiponectin was increased in mice treated with CuE while leptin levels were reduced in the serum (Fig 4E and 4F). Obesity induced inflammation and subsequent cytokine release has central role in impairing the fat tissue function [28]. The degree of inflammation in fat tissue was measured by expression of macrophage marker gene, *CD11b*, monocyte chemoattractant protein-1*(MCP-1)* and its receptor the c-c chemokine recptor type 2 *(CCR2)*. The expression of all these genes was reduced in visceral fat of mice treated with CuE (Fig 4G–4I). We also observed reduced TNF-alpha expression after treatment of mice with CuE (Fig 4J).

### Evaluation of CuE induced mechanism to modulate adipose tissue biology

**Fig 5:**
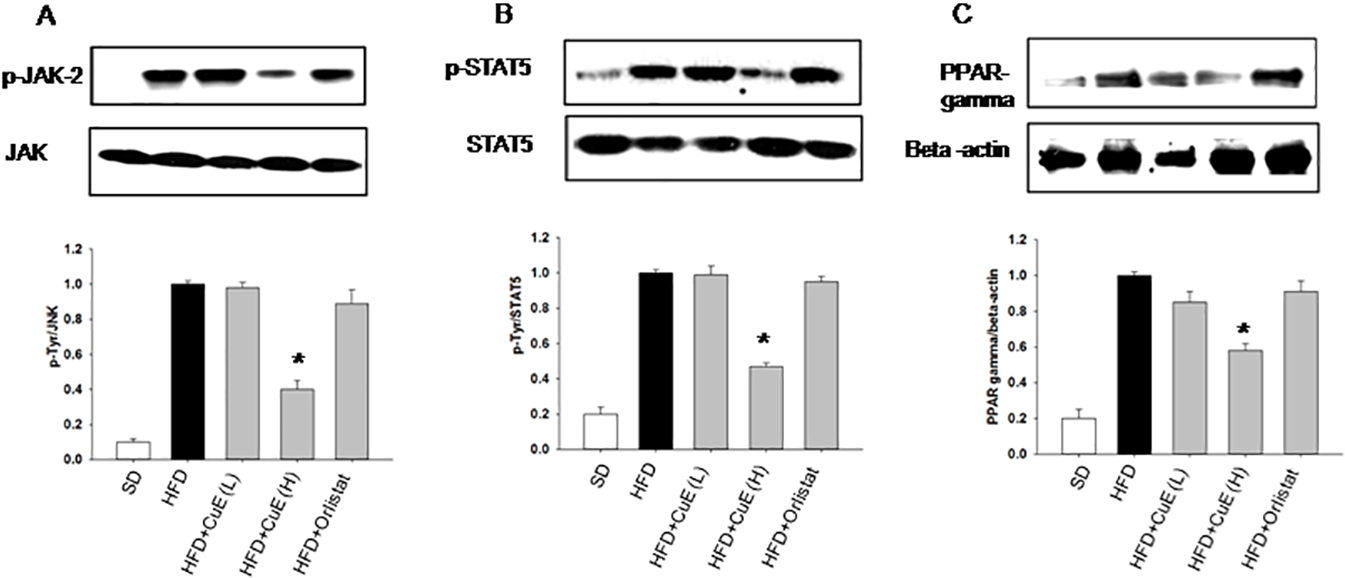
Determination of the effect of CuE on JAK-STAT signaling. (A) Total protein from abdominal fat of all mice groups was separated on 7.5% SDS-PAGE gels, and immunoblotted with a phospho-tyrosine1007/1008 antibody. The same blots were stripped and reprobed with a polyclonal JAK-2 protein antibody. The levels of tyrosine phosphorylation of JAK-2 in the immunoblots were quantified using densitometry and normalized to the JAK-2 protein. (B) Phospho STAT5A tyrosine 694 antibody was used for immunoblotting STAT5A phosphorylation. (C) PPAR-gamma antibody was used to measure expression of PPAR-gamma. The data are presented as mean ± SEMs, n = 5–6, *P < 0.05 mice treated with CuE or Orlistat vs HFD-MetS mice.

First, the effect of CuE on different STAT proteins was evaluated; reduction in STAT5 activity was observed after CuE treatment, however, phosphorylation levels of STAT1 and STAT3 remained unchanged (S2 Fig). We targeted effect of CuE on JAK-STAT signaling in adipose tissue. In visceral fat, CuE treatment attenuated Jenus kinase (JAK) activity by reducing its tyrosine phosphorylation (Fig 5A). STATs are the key substrates of JAK. We observed reduced STAT-5 phosphorylation in abdominal fat of mice treated with CuE (Fig 5B). PPAR-gamma is a master regulator of adipogenesis [29] and since PPAR gamma is directly affected by STAT-5 activity [30], we found reduction in PPAR-gamma expression after CuE treatment (Fig 5C). This result indicates mechanism of CuE in improving adipose tissue dysfunction.

### Effect of CuE on insulin resistance

**Fig 6:**
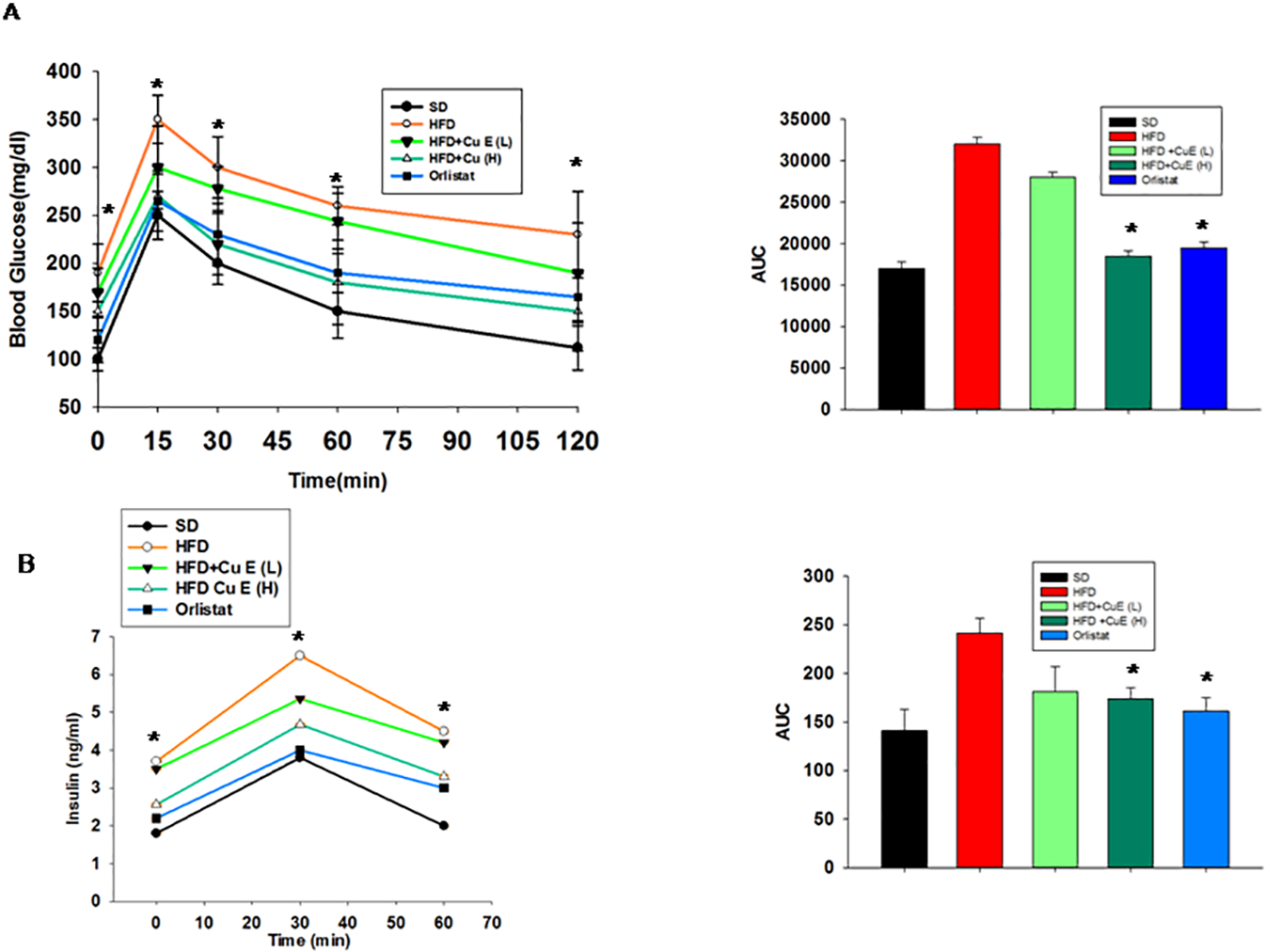
CuE treatment improved insulin resistance in mice. (A) Measurement of blood glucose levels in mice when challenged with intraperitoneal glucose tolerance test (IP-GTT). Area under the curve (AUC) quantification for GTT. (B) Measurement of blood insulin levels during IP-GTT. Area under the curve (AUC) quantification for glucose stimulated insulin secretion (GSIS). Results represent mean ± SEMs. n = 10–12 in each group, *P < 0.05, mice treated with CuE or Orlistat vs HFD mice model of MetS.

Insulin resistance is the key abnormality associated with metabolic syndrome [31]. We asked the question whether CuE has the potential to improve insulin resistance in mice model of MetS. We found that CuE treatment improved glucose intolerance in HFD-MetS mice (Fig 6A). In addition to increased glucose absorption, we observed CuE treated mice showed reduced basal insulin levels and maintained reduced insulin secretion during glucose challenge as compared to the HFD-MetS mice (Fig 6B).

### Determination of CuE mediated molecular mechanism to improve insulin action in muscle tissue

**Fig 7:**
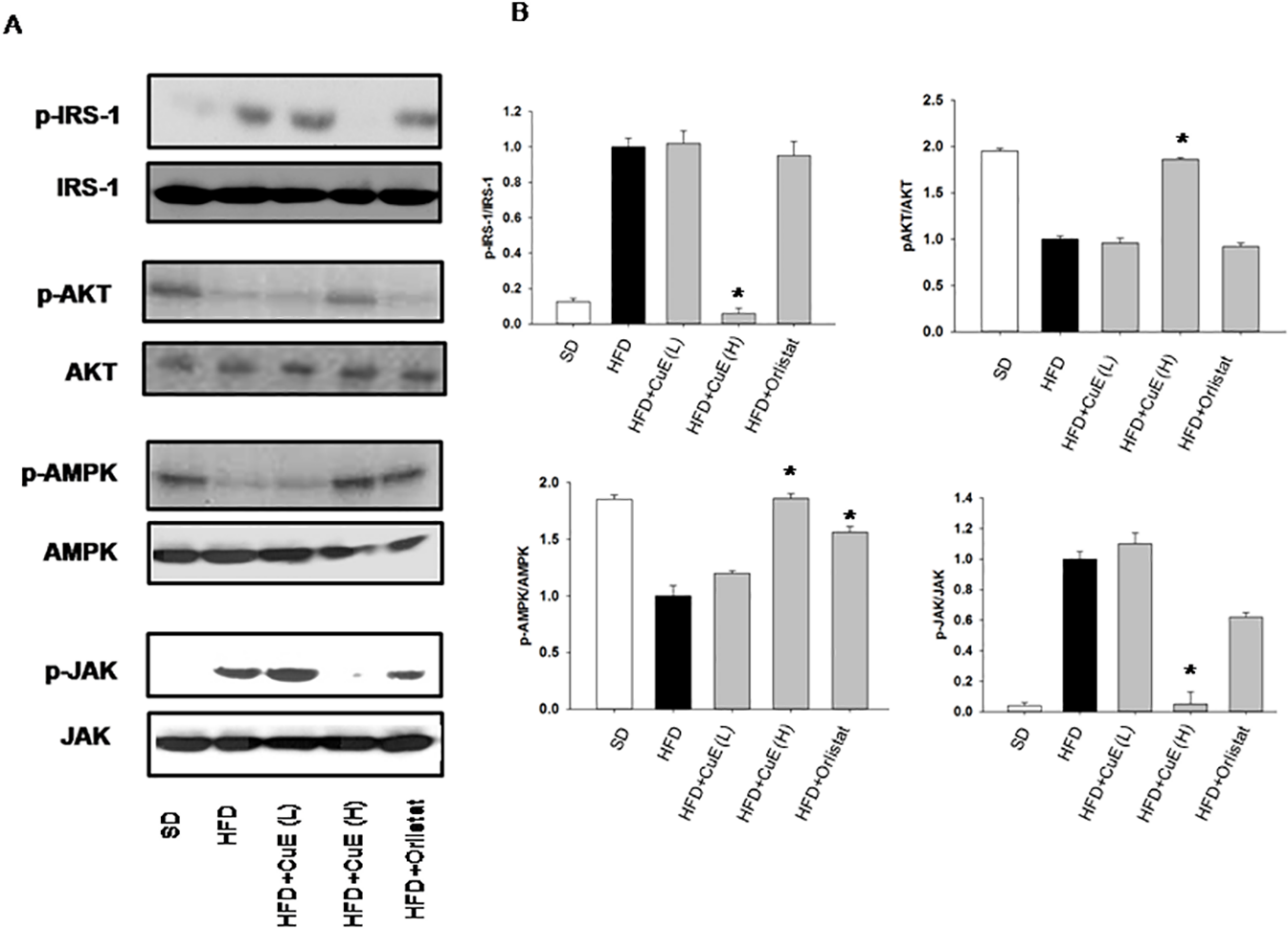
Determination of the effect of CuE on insulin signaling. (A) Total protein from skeletal muscle of all mice groups was separated on 7.5% SDS-PAGE gels, and immunoblotted with either phospho IRS-1 serine 307 or phospho AKT serine 473 or phospho-AMPK-Thr 172 or phospho JAK-tyrosine1007/1008 antibody. (B) The levels of phosphorylation in the immunoblots were quantified using densitometry and normalized to their respective total proteins expression. The data are presented as mean ± SEMs, n = 5–6, *P < 0.05 mice treated with CuE or Orlistat vs HFD-MetS mice.

First, we targeted CuE effect on insulin signaling. We observed that IRS-1 serine phosphorylation levels diminished in muscle tissue of mice when injected with intravenous insulin (Fig 7A). Subsequently, there was also enhanced activity of PKB in muscle tissue (Fig 7A). In order to decipher the mechanism behind CuE mediated up-regulation of insulin signaling, JAK-STAT axis was investigated in muscle tissue. We observed reduced JAK-2 phosphorylation in mice treated with CuE (Fig 7A and 7B). STAT5 activity was reduced in muscle; however results were not statistically significant (S3 Fig). A recent report indicated CuE mediated autophagy by enhancing AMP-activated protein kinase (AMPK) activity [32]. Significant increase in phosphorylation of AMPK in mice muscle was observed after CuE treatment (Fig 7A and 7B).

### Assessment of other metabolic parameters in mice model of MetS

Table 1 indicates the blood levels of free fatty acids (FFA), triglycerides, cholesterol, and low density lipoproteins (LDL). The concentrations of FFA, triglycerides, LDL and cholesterol were significantly increased initially in HFD-MetS mice, however, these levels were significantly reduced after treatment with CuE.

**Table 1:** Serum lipid levels in experimental mice.

	TG (mg/dl)	LDL (mg/dl)		Cholesterol (mg/dl)	Free Fatty Acids (mg/dl)
SD	74.78 ± 6.8		40.04 ±2.6	99.67 ± 4.4	62.35 ± 2.8
HFD	192.27 ± 10.2		156.52 ±12.5	170.00 ± 6.9	186.24 ± 9.8
HFD+CuE (L)	144.33 ± 9.5		124.30 ± 5.6	129.25 ±3.7	150.51 ± 6.0
HFD+CuE (H)	85.14* ± 5.2		50.40* ± 3.0	101.75* ± 2.9	78.02* ± 4.6
HFD+Orlistat	80.00* ± 4.1		40.85* ± 2.5	95.00* ± 5.6	75.65* ± 2.7

The data are presented as means ± SEMs, n = 5–6

*P < 0.05 mice treated with CuE vs HFD-MetS mice.

## Discussion

Adipocyte play pivotal role in energy balance and metabolism [33]. In the present investigation, we targeted fat tissue biology and dysfunction to treat metabolic syndrome. Accumulating evidence indicates anti-proliferative, anti-inflammatory and autophagic properties of Cucs and CuE in particular [12, 34, 35]. However, role of cucrbitacins in adipose tissue biology is poorly defined [16]. This study presents evidence of CuE mediated suppression of obesity and insulin resistance in MetS mice for the first time. We observed inhibition of JAK and specifically STAT5 transcriptional factors by cucurbitacin E in adipose tissue of mice model of MetS (Fig 5A and 5B). Among all STAT proteins, the physiological relevance of STAT5 in adipogenesis is widely studied [36] [37] [38]. CuE mediated improvement of adipokine such as adiponectin and inhibition of inflammatory parameters from adipose depots was also observed (Fig 4E and 4J). Obesity induced hyperleptinemia was found to have been diminished by CuE treatment in mice model of MetS (Fig 4F). CuE also affected *de novo* lipogenesis, as indicated by attenuation of lipogenic gene expression and plasma lipids levels after CuE treatment (Fig 4A and 4B and Table 1). Since we observed CuE mediated inhibition of JAK in fat tissue, we hypothesized that CuE may enhance insulin signaling by inhibiting JAK in muscle tissue of mice. Our results verify the inhibition of JAK-2 and enhanced activity of AMPK in muscle tissue of MetS mice model (Fig 7A and 7B). CuE mediated, reduced tendency of STAT5 phosphorylation in muscle (S3 Fig) indicated consistency with reduced STAT-5 expression in fat tissue. CuE was also found to have upregulated insulin signaling as indicated by diminished serine phosphorylation of IRS-1 and enhanced activity of PKB. Previously, several studies revealed JAK mediated enhanced serine phosphorylation of IRS-1 [39] [40]. Furthermore, GTT, GSIS measurements and reduction in hyperinsulinemia corroborated the potential of CuE to reduce insulin resistance (Fig 6A and 6B). AMPK mediated metabolic effects of insulin in skeletal muscle are well reported [41], therefore, we suggest that improvement in insulin sensitivity by CuE is partly explained by activation of AMPK (Fig 7A and 7B). In conclusion, CuE, in low doses, attenuated central obesity, dyslipedima and insulin resistance in mice model of MetS. Therefore, we propose cucurbitacin E as a new therapeutic target for improving pathophysiology of MetS and related diseases.

## Supporting information

S1 Fig 3T3-L1 adipocytes were treated with different concentrations of cucurbitacins (for 72 hrs). Percentage viability of the cells as compared to control cells. (Mean ± SEMs, n = 5, *p < 0.05 cells stimulated with cucurbitacins vs. non stimulated cells).
(DOCX)

S2 Fig Determination of the effect of CuE on STAT proteins activation. (A) Total protein from abdominal fat of all mice groups was separated on 7.5% SDS-PAGE gels, and immunoblotted with phospho STAT1, phosphor STAT3 and phosphor STAT5. The same blots were stripped and reprobed with a respective STAT protein antibody.
(DOCX)

S3 Fig Determination of the effect of CuE on STAT-5 activation in muscle tissue. (A) Total protein from muscle tissue of all mice groups was separated on 7.5% SDS-PAGE gels, and immunoblotted with phospho STAT5. The same blots were stripped and reprobed with a respective STAT protein antibody.
(DOCX)
