# Supplementary material for: Cucurbitacin E reduces obesity and related metabolic dysfunction in mice by targeting JAK-STAT5 signaling pathway
Source: PLoS One. 2017 Jun 9;12(6):e0178910. doi: 10.1371/journal.pone.0178910 (PMC5466318; doi:10.1371/journal.pone.0178910)
Supplement: S1 Fig — Percentage viability of the cells as compared to control cells. (Mean ± SEMs, n = 5, *p < 0.05 cells stimulated with cucurbitacins vs. non stimulated cells). (DOCX) [file pone.0178910.s001.docx]

**Supporting information**

**
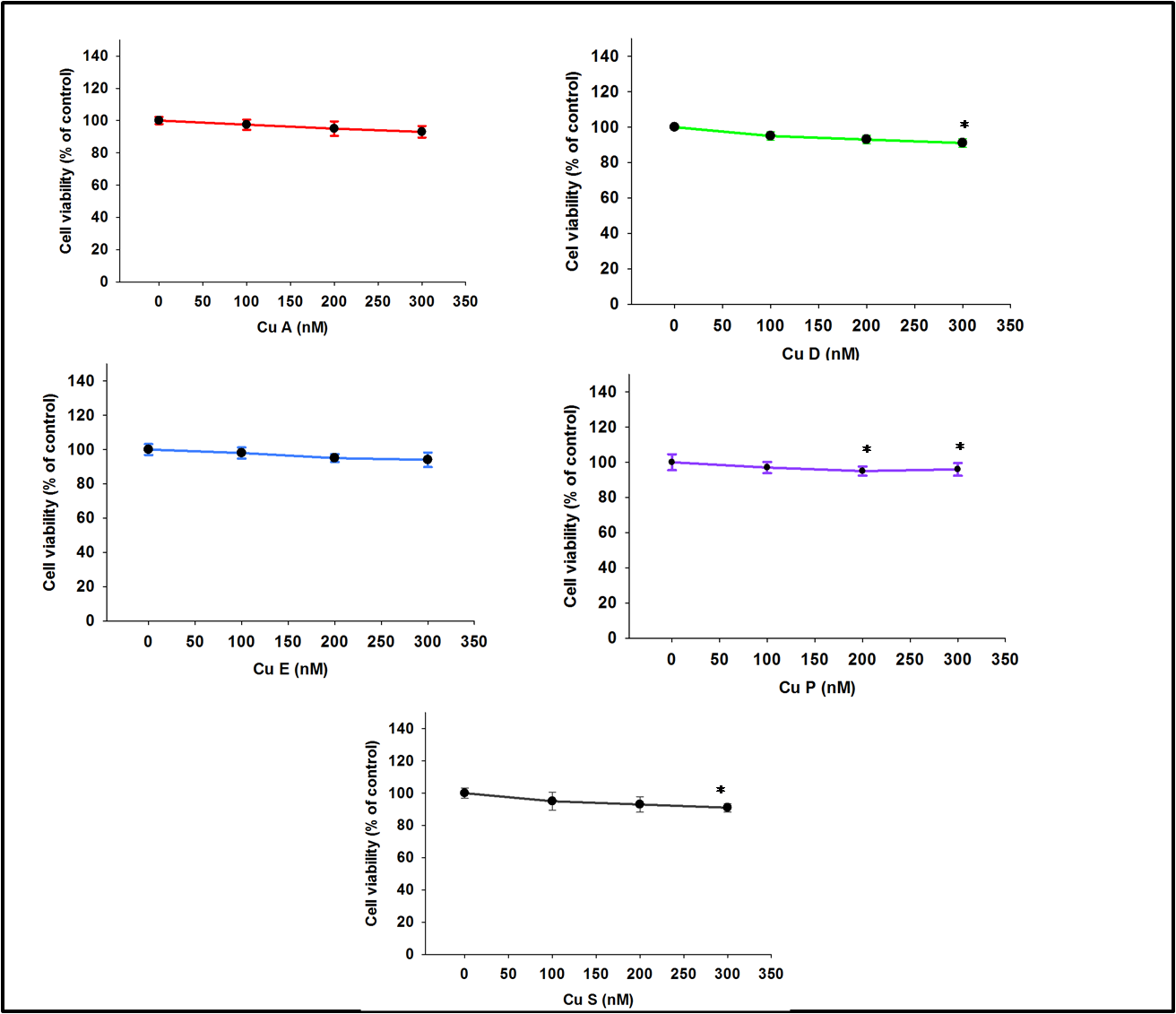
**

**S1 Fig..** 3T3-L1 adipocytes were treated with different concentrations of cucurbitacins (for 72 hrs). Percentage viability of the cells as compared to control cells. (Mean ± SEMs, n = 5, *p < 0.05 cells stimulated with cucurbitacins vs. non stimulated cells).
