# Supplementary material for: Cucurbitacin E reduces obesity and related metabolic dysfunction in mice by targeting JAK-STAT5 signaling pathway
Source: PLoS One. 2017 Jun 9;12(6):e0178910. doi: 10.1371/journal.pone.0178910 (PMC5466318; doi:10.1371/journal.pone.0178910)
Supplement: S3 Fig — (A) Total protein from muscle tissue of all mice groups was separated on 7.5% SDS-PAGE gels, and immunoblotted with phospho STAT5. The same blots were stripped and reprobed with a respective STAT protein antibody. (DOCX) [file pone.0178910.s003.docx]

**Supporting information**

**
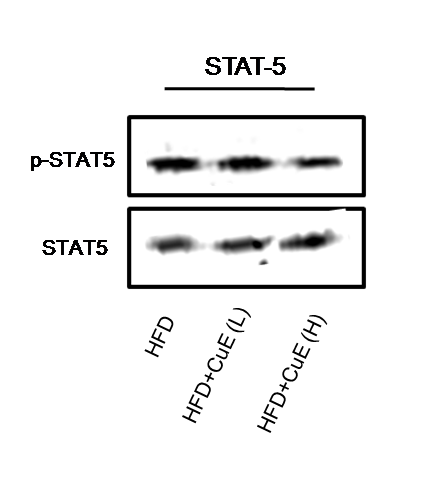
**

**S3 Fig. Determination of the effect of CuE on STAT-5 activation in muscle tissue.** (A) Total protein from muscle tissue of all mice groups was separated on 7.5% SDS-PAGE gels, and immunoblotted with phospho STAT5. The same blots were stripped and reprobed with a respective STAT protein antibody.
